# Outbreak of MRSA in a Gynecology/Obstetrics Department during the COVID-19 Pandemic: A Cautionary Tale

**DOI:** 10.3390/microorganisms10040689

**Published:** 2022-03-23

**Authors:** Mareike Möllers, Marie-Kristin von Wahlde, Franziska Schuler, Alexander Mellmann, Christian Böing, Vera Schwierzeck, Julia Sophie Schneider, Stefanie Kampmeier

**Affiliations:** 1 Department of Obstetrics and Gynecology, University Hospital Münster, 48149 Münster, Germany; mareike.moellers@ukmuenster.de (M.M.); marie-kristin.vonwahlde@ukmuenster.de (M.-K.v.W.); 2 Institute of Medical Microbiology, University Hospital Münster, 48149 Münster, Germany; franziska.schuler@ukmuenster.de; 3 Institute of Hygiene, University Hospital Münster, 48149 Münster, Germany; alexander.mellmann@ukmuenster.de (A.M.); christian.boeing@ukmuenster.de (C.B.); vera.schwierzeck@ukmuenster.de (V.S.); julia.schneider@ukmuenster.de (J.S.S.)

**Keywords:** MRSA, surgical site infection, COVID-19, infection control, outbreak management, contact precautions

## Abstract

Since March 2020, the COVID-19 pandemic forced hospitals worldwide to intensify their infection control measures to prevent health care-associated transmission of SARS-CoV-2. The correct use of personal protective equipment, especially the application of masks, was quickly identified as priority to reduce transmission with this pathogen. Here, we report a nosocomial cluster of methicillin-resistant *Staphylococcus aureus* (MRSA) that occurred during the COVID-19 pandemic in a gynecology/obstetrics department, despite these intensified contact precautions. Five MRSA originating from clinical samples after surgical intervention led to an outbreak investigation. Firstly, this included environmental sampling of the operation theatre (OT) and, secondly, a point prevalence screening of patients and health care workers (HCW). All detected MRSA were subjected to whole genome sequencing (WGS) and isolate relatedness was determined using core genome multilocus sequence typing (cgMLST). WGS revealed one MRSA cluster with genetically closely related five patient and two HCW isolates differing in a single cgMLST allele at maximum. The outbreak was terminated after implementation of infection control bundle strategies. Although contact precaution measures, which are also part of MRSA prevention bundle strategies, were intensified during the COVID-19 pandemic, this MRSA outbreak could take place. This illustrates the importance of adherence to classical infection prevention strategies.

## 1. Introduction

Severe acute respiratory syndrome coronavirus 2 (SARS-CoV-2) was detected in Wuhan City, China, in December 2019. In March 2020, the WHO declared the COVID-19 disease a pandemic. Soon after SARS-CoV-2 spread to Germany and forced the hospitals to increase their infection prevention and control (IPC) measures. These included physical distancing, visitor restrictions and contact precautions, which in particular comprised the use of protective personal equipment (PPE) such as surgical masks. As pregnant women and cancer patients are considered a risk group for severe COVID-19 outcomes, the implementation of SARS-CoV-2 infections IPC measures was a priority for gynecology/obstetrics departments in Germany [1].

As contact precautions are part of infection prevention bundle strategies also established in the context of prevention of multi-drug-resistant organisms (MDRO), i.e., methicillin-resistant *S. aureus* (MRSA) or vancomycin-resistant enterococci (VRE), one would expect a decrease of hospital-acquired MDRO during the COVID-19 pandemic. In this context, a study from Los Angeles, USA, reported a decline in the MDRO rate per 1000 patients between Q1 and Q2 2020 of 41% for MRSA, 80% for VRE [2]. On the other hand, an increase in antimicrobial resistances during the COVID-19 pandemic has been observed and is summarized in a review from Taiwanese investigators [3] and in an observational study performed in Brazil [4]. Additional outbreak reports dealing with clusters of VRE or multi-resistant gram-negative bacteria [5,6] indicate the possibility of enhanced MDRO acquisition during the pandemic situation.

In recent years, MRSA prevalence in critically ill patients in Germany has decreased, but MRSA is still one of the most common MDRO causing hospital-acquired infections also in obstetrics and gynecology patients [7,8]. As it is usually found in the nasal vestibule, one bundle strategy for MRSA prevention usually includes usage of facemasks also reflecting a main component of SARS-CoV-2 transmission prevention.

Nevertheless, here, we present results of an MRSA outbreak we experienced in our department of obstetrics and gynecology in summer 2020.

## 2. Materials and Methods

### 2.1. Clinical Setting and Infection Control Measures

The University Hospital Münster (UHM) is a 1500-bed care center admitting 55,000 patients per year. In accordance with national infection prevention recommendations [9], all patients in our hospital are screened for MRSA on admission. Standard procedure for MRSA positive patients includes decolonization of patients and contact precautions comprising patient isolation in a single room with separate sanitary facilities and the use of personal protective equipment (gloves, gowns and surgical facemasks). Isolation of patients can be discontinued if three negative series of swab samples (nose/throat, axilla/groin, wounds where appropriate) are collected. HCW are not tested routinely. If tested positive for MRSA, HCW have to undergo decolonization treatment and are employed distantly to patients until further notice. Return to usual work is possible if three negative series of swab samples are collected.

During the COVID-19 pandemic, masks (surgical facemasks or FFP2 masks) were worn by employees, visitors and, if possible, for health reasons, also by patients. Hence, a main component of MRSA transmission prevention was established due to the pandemic situation.

Nevertheless, in June 2020, we faced an increase of MRSA isolated from clinically relevant samples of patients on a gynecology/obstetric ward during a three-month-timespan (Table 1. In total, five patients acquired an infection after their initial screening for MRSA on admission was negative. Therefore, these infections were classified as hospital-acquired. As already two infections occurring in June exceeded the baseline infection rate of one MRSA infection per year on this ward, we initiated an outbreak investigation in July 2020 comprising epidemiological research, environmental swab sampling to detect contaminations of patient surroundings and a point prevalence screening of all patients and involved HCW on the ward and in the operation theatre (OT). Additionally, hand hygiene training was performed among HCW. Intensified surface disinfection was established using an alkylamine, IncidinTM plus 0.5% (ECOLAB Healthcare, Monheim am Rhein, Germany) comprising patient rooms, nurses’ rooming homes and storage rooms.

### 2.2. MRSA Culturing and Typing of Screening Samples

Detection of MRSA was performed by using selective agar plates (chromID, bioMérieux, Marcy l’Étoile, France, 36 ± 1 °C for 24 h). For species identification, we used MALDI-TOF mass spectrometry (Bruker Daltonics, Bremen, Germany). Susceptibility testing (see also Table 2) was performed using the VITEK-2 system (bioMérieux) and interpreted according to the European Committee on Antimicrobial Susceptibility Testing (EUCAST) standards for clinical breakpoints (version 11.0). Isolates were further screened by PBP2a (PBP2a SA Culture Colony Test, Abbott, Scarborough, Maine, USA) and, in case of inconsistent results, for *mecA*, *mecC* and panton-valentine leukocidin (PVL) (eazyplex^®^ MRSAplus, amplex, Gars-Bahnhof, Germany).

### 2.3. Environmental Sampling and Testing Method

Environmental sampling was performed using sterile packaged polywipes (mwe, Corsham, Wiltshire, UK) on contact surfaces and incubating them in Tryptic Soy Broth + lecithin tween (LT) (Merck Millipore, Eppelheim, Germany) at 37 °C for 24 h. Ten μL of this broth were streaked onto blood agar and MRSA selective agar and incubated at 37 °C for 24 h. Suspected colonies were subcultured on blood agar and species identification was performed by MALDI-TOF mass spectrometry. Susceptibility testing was performed via agar diffusion and evaluated in accordance with the EUCAST standards for clinical breakpoints (version 11.0).

### 2.4. Whole Genome Sequencing-Based Typing

For genetic comparison, confirmed *S. aureus* isolates were subjected to whole genome sequencing (WGS)-based typing using either the MiSeq platform (Illumina Inc., San Diego, CA, USA) or the Sequel II platform (Pacific Biosciences Inc., Menlo Park, CA, USA). The Nextera XT protocol (Illumina Inc.) was used to prepare genomic DNA for MiSeq sequencing. The resulting 250 bp paired-end reads were then de novo-assembled after quality-trimming using the SKESA algorithm [10] using default parameters of the SeqSphere+ software version 7.0.1 (Ridom GmbH, Münster, Germany). For Pacific Biosciences sequencing, we constructed the sequence library using the SMRTbell Express Template Prep Kit 2.0 (Pacific Biosciences Inc.) in accordance to the manufacturer’s recommendations. The resulting reads were then assembled applying the “Microbial Assembly” pipeline within the SMRT Link software version 9 (Pacific Biosciences Inc.) using default parameters except for the genome size, which was adopted to 2.8 Mb. For all WGS datasets, we utilized SeqSphere+ to compare coding regions in a gene-by-gene approach, i.e., cgMLST as described previously [11], using the public cgMLST scheme for *S. aureus* [12]. Clonal relationship of genotypes is displayed using a minimum spanning tree algorithm calculated by the same software and is rated as closely related if genotypes differ in ≤6 alleles. Multilocus sequence typing (MLST)- and *spa*-genotypes as well as the presence of virulence and resistance genes, i.e., *lukS*, *lukF*, *mecA* and *mecC*, were extracted from the WGS data in silico.

### 2.5. Ethics Statement

All strategies and investigations were performed in accordance with the national recommendations for outbreak investigations of the German legally assigned institute for infection control and prevention (Robert-Koch Institute). Formal consent was therefore not required.

## 3. Results

### 3.1. Outbreak Management

Epidemiological investigations uncovered that all patients suffering from MRSA infections during this outbreak, had undergone a surgical procedure in the same OT. Hence, after notification of the outbreak to public health authorities, environmental sampling focused on surfaces in this OT and the associated sterile good storage resulting in 25 sampling probes (Table 3). Of these, four were positive for *S. aureus* but lacked methicillin-resistance. In all other probes, only microorganisms of skin flora and the environment were detected (Table 3).

Point prevalence screening revealed no additional MRSA colonized patients on ward. In contrast, five MRSA colonized HCWs could be detected during the screening efforts.

After establishing the previously mentioned infection control bundle strategy (i.e., training of HCWs, intensified disinfection, surveillance sampling), the cluster could be terminated in September 2020. No further MRSA infections nor hospital-acquired colonizations occurred until the day of writing.

### 3.2. Whole Genome Sequencing-Based Typing

**Figure 1:**
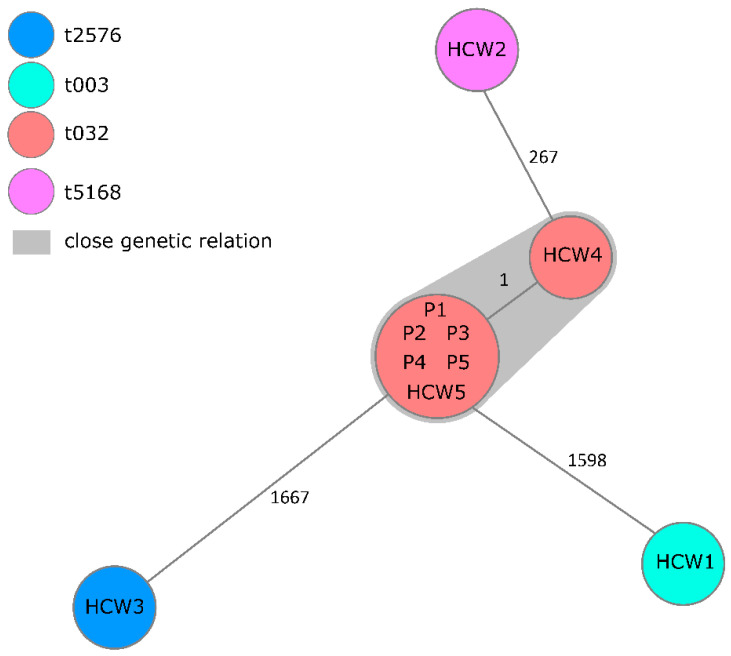
Minimum spanning tree of detected MRSA in chronological order. Minimum spanning tree of five healthcare worker (HCW) and five patient (P) isolates illustrating their genotypic relationship based on up to 1861 cgMLST target genes, pairwise ignore missing values. Every circle represents one genotype, the size of circles correlates with the number of identical genotypes. Color of circles indicates MRSA spa-type. Grey coloring indicates close genetic relation.

All ten MRSA isolates (five originating from patients and HCW each) were subjected to WGS. The analysis resulted in one cluster of seven genetically closely related, i.e., one cgMLST allele distance only, or identical strains, comprising genotypes from all patients and two HCW as well as three singletons with no genetic relation to this cluster. In all isolates, *mecA* gene could be detected in absence of *mecC* and *lukS* or *lukF* indicating no PVL existence. Most prevalent *spa*-type was t032 in all strains of the cluster, whereby t003, t2576 and t5168 were found in one isolate each (Figure 1).

## 4. Discussion

Transmissions of MRSA in the clinical setting can occur via direct and indirect contact [9,13] mostly originating from the nasal vestibule, which is the main reservoir for this pathogen in humans. Thus, up to now, contact precautions, i.e., wearing of masks are part of infection control bundle strategies preventing the spread of MRSA.

Despite these intensified measures including wearing of masks that were established from the very beginning of the COVID-19 pandemic, an MRSA outbreak could take place on a gynecology/obstetrics ward in our tertiary care center. This leads to the question if adherence to the infection control bundle strategies for MRSA in the hospital and public health setting during COVID-19 are appropriate. Our study suggests that especially medical staff do not adhere to so-called “basic hygienic measures” despite an increased awareness concerning personal protective equipment (PPE) during the COVID-19 pandemic. Lack of adequate hand hygiene adherence and inadequate use of PPE have been highlighted by recent studies to be causative for multi-drug-resistant organism (MDRO) outbreaks during COVID-19 [14]. Interestingly, during the SARS-CoV epidemic in 2002–2003, a similar MRSA outbreak was reported [15]. In this, infection control measures, such as constant wearing of gloves, gowns and masks, were implemented in an intensive care unit (ICU) to maximize staff protection. This practice, along with other factors, led to a significantly increased acquisition of MRSA in patients despite a low importation rate of MRSA into the ICU. One possible explanation of this observation is that staff awareness for basic hygiene measures, i.e., hand hygiene, decreased, as staff had to adjust to wear PPE required to prevent droplet transmission during the pandemic.

Our outbreak report illustrates the importance of WGS-based typing as part of a routine surveillance strategy for the hospital setting and public health-related questions. WGS is an effective method to detect MRSA transmissions over several weeks and without direct patient-to-patient contact of MRSA colonized or infected patients. With other common typing methods, i.e., *spa*-typing, this outbreak would have probably been not discovered and contained as the *spa*-type t032 identified is one of the most prevalent *spa*-types in our local area. Implementation of routine WGS typing of MRSA and other MDRO, including VRE and gram-negative MDRO should therefore be more widely used.

During the COVID-19 pandemic period, only outbreaks involving VRE and gram-negative bacteria have been published until today, most of them occurring in intensive care units (ICU) [5,16]. So far, there have been no MRSA outbreak reports linked to the COVID-19 pandemic. Possible explanations for these observations are diverse. On the one hand, there is an increased rate of antimicrobial agent utilization in ICU settings [3,4], notably affecting selection of gram-negative bacteria; on the other hand, especially VRE have an increased tenacity on inanimate surfaces compared to staphylococci [17,18] and can thereby easier be source of transmissions. Contradictory to these findings, more recent studies related to the current pandemic show a decrease of MDRO infections, which is explained as an unintentional side effect of the large-scale use of infection prevention measures during the pandemic [19,20] but which can also result from a decrease in the total number of patients tested [21,22].

## 5. Conclusions

Adherence to basic hygiene measures is essential to prevent the transmission of MRSA in the clinical context. Additionally, basic hygiene measures should not be neglected, despite the contact precautions established during the COVID-19 pandemic. WGS-based approaches can thereby help to early detect and terminate hospital transmissions of MDR bacteria.

## Figures and Tables

**Table 1:** Clinical characteristics of methicillin-resistant *S. aureus* (MRSA) infected patients.

Patient No.	Surgical Intervention	MRSA Screening Date and Result	Clinical MRSA Sample	Sampling Date	Hospital- Acquired
P1	Curettage	12 June 2020 negative	Vaginal swab sampling	27 June 2020	Yes
P2	Hysterectomy	26 May 2020 Negative	Drain fluid	29June 2020	Yes
P3	Plastic surgery of the breast	6 July 2020 Negative	Pus from breast tissue	13 July 2020	Yes
P4	Cesarean section	8 June 2020 negative	Surgical wound swab	6 August 2020	Yes
P5	Laparotomy	16 May 2020 negative	Swab sampling of the port puncture site	21 September 2020	Yes

**Table 2:** Susceptibility testing results of methicillin-resistant *S. aureus* (MRSA) detected in patients.

No.	P	OX	AMX/CA	CEZ	LEV	CLI	VAN	TEI	TMP/SMX	RIF	FOS	LIN	DAP	MUP
P1	R	R	R	R	R	R	S	S	S	R	R	S	R	I
P2	R	R	R	R	R	R	S	S	S	S	S	S	S	S
P3	R	R	R	R	R	R	S	S	S	S	S	S	S	S
P4	R	R	R	R	R	R	S	S	S	S	S	S	S	S
P5	R	R	R	R	R	R	S	S	S	S	S	S	S	I

P—penicillin; Ox—oxacillin; AMX/CA—amoxicillin/clavulanic acid; CEZ—cefazolin; LEV—levofloxacin; CLI—clindamycin; VAN—vancomycin; TEI—teicoplanin; TMP/SMX—trimethoprim/sulfamethoxazole; RIF—rifampicin; FOS—fosfomycin; LIN—linezolid; DAP—daptomycin; MUP—mupirocin.

**Table 3:** Environmental swab sampling.

Sampling Site	Detected Microorganisms
Preparation room, work space	*Bacillus* spp., CNS
Preparation room, storage	*Bacillus* spp., CNS
Preparation room, storage	*Bacillus* spp.
Preparation room, storage	*Bacillus* spp.
Preparation room, storage	*Bacillus* spp., CNS
Operation room, PC keyboard	*Bacillus* spp., *S. aureus*
Operation room, PC keyboard	*Bacillus* spp., CNS
Operation room, perfusor	*Bacillus* spp., *S. aureus*
Operation room, PC monitor	*Bacillus* spp., CNS
Operation room, supply trolley	*Bacillus* spp.
Operation room, infusion stand	*Bacillus* spp., CNS
Operation room, instruments table	*Bacillus* spp., CNS
Operation room, suture storage	*S. aureus,* viridans streptococci
Operation room, leg support	*Bacillus* spp., CNS
Operation room, remote control	*Bacillus* spp., *S. aureus*
Operation room, disinfection bottle	*Bacillus* spp., CNS
Operation room, disposable gloves	*Bacillus* spp.
Operation room, disinfection bottle	*Bacillus* spp., CNS
Sterile goods storage, shelves	*Bacillus* spp., CNS
Sterile goods storage, shelves	*Bacillus* spp., CNS
Sterile goods storage, shelves	*Bacillus* spp., CNS
Sterile goods storage, operation set	*Bacillus* spp., CNS
Sterile goods storage, operation set	*Bacillus* spp., CNS

CNS—coagulase negative staphylococci.

## Data Availability

Whole genome sequences of all MRSA isolates are available at NCBI GenBank under BioProject accession number PRJNA808065.
